# Pro-inflammatory cytokine-driven molecular signaling in skeletal muscle during cancer cachexia

**DOI:** 10.1042/BST20260486

**Published:** 2026-05-15

**Authors:** Benudhara Pati, Nibedita Nandy, Birendra Kumar Bindhani, Naresh Chandra Bal

**Affiliations:** School of Biotechnology, KIIT University, Bhubaneswar, Odisha 751024, India

**Keywords:** adipocytes, Cancer cachexia, JAK/STAT3 pathway, Muscle wasting, NF-κB pathway, Pro-inflammatory cytokines

## Abstract

Cancer cachexia is a multifactorial syndrome characterized by the progressive loss of muscle and fat, commonly observed among patients with cancer. It is very distinct from other skeletal muscle wasting such as sarcopenia and malnutrition and is known to reduce cancer treatment effectiveness. Cachexia progression is driven by a combination of factors, including hormonal dysregulation, anorexia, tumor-derived catabolic factors (in cancer cachexia), and systemic or muscular inflammation, all of which worsen overall muscle health. In this review, we will probe the role of pro-inflammatory cytokines, such as IL-6, IFN-γ, TNF-α, TGF-β, IL-1β, and IL-8, in driving the systemic inflammation and disruption of muscle metabolic homeostasis that support the development of cachexia. These cytokines may be produced from various organs, including the adipose depots that contribute to muscle wasting and metabolic dysfunction by disrupting the equilibrium between anabolic and catabolic processes. The ubiquitin-proteasome system, NF-κB, and JAK/STAT3 are important molecular pathways that mediate cytokine-induced catabolic signaling. The review further analyzes the context-dependent dual functions of these cytokines and the molecular mechanisms underlying the loss of their regulatory control during cancer progression. The limited success of current therapeutic approaches for cancer cachexia highlights the urgent need for evaluation of more targetable mechanisms for the treatments. Here, one of our main objectives is to probe whether suppression of pro-inflammatory cytokine signaling and activation of anti-inflammatory pathways can be utilized to modulate the tumor microenvironment, thereby countering cancer cachexia.

## Introduction

Cancer cachexia is a multifactorial syndrome, which was defined by the international consensus of 2011 as loss of skeletal muscle mass, with or without loss of fat mass, that cannot be fully reversed by conventional nutritional support and leads to progressive functional impairment and reduced treatment tolerance. However, “cachexia (or, wasting syndrome)” is a complex metabolic condition characterized by the consistent loss of skeletal muscle mass, which is frequently coupled with a loss of body fat [1,2]. It is commonly associated with chronic diseases such as heart failure (cardiac cachexia), obstructive pulmonary disease (pulmonary cachexia), kidney disease (renal cachexia), diabetes (diabetic cachexia), and rheumatoid arthritis (rheumatoid cachexia) [3–7]. Though it predominantly affects muscular tissue, the entire body is affected by cachexia and frequently coexists with age-linked disorders like sarcopenia and frailty. Early detection of the illness is challenging since muscle loss continues even with a healthy diet. Once symptoms appear, cachexia leads to progressive weight loss, reduced energy, appetite loss, and physical decline, which cannot be reversed by nutrition. A number of factors, including decreased food intake, hormonal dysregulation, and chronic inflammation, contribute to this metabolic imbalance [8–10]. This systemic condition emphasizes how critical it is to view cachexia as a widespread metabolic syndrome that requires specialized care and intervention rather than as a problem affecting a single organ system. Cachexia is more challenging to treat than sarcopenia or malnutrition since it is often associated with systemic inflammation and does not respond to conventional nutritional therapy [11].

Historically, there has been little attention on accurately diagnosing, classifying, and staging cachexia, as the clinical focus has generally been on later stages of disease. However, consensus has evolved on three-stage model for cancer-related wasting: pre-cachexia, cachexia, and refractory cachexia [12]. Pre-cachexia is defined by weight loss of less than 5%, mild metabolic alterations, appetite loss, fatigue, and low-level inflammation, providing an opportunity for early intervention through dietary support and monitoring. Cachexia is characterized by greater than 5% body weight loss, severe muscle wasting, fatigue, appetite loss, and systemic inflammation, necessitates dietary, medical, and psychological support. Refractory cachexia, the final stage, occurs when treatments are no longer effective or acceptable as the condition progresses. Patients at this stage have considerable weight loss (>15% body weight loss with a body mass index (BMI) <23 kg/m or >20% weight loss with a BMI <27 kg/m), intense exhaustion, and a high symptom burden; therefore, palliative care is the goal [13]. Aggressive nutritional intervention is usually terminated in favor of symptom treatment and psychosocial support. This structured staging system enables timely detection, personalized therapy planning, and better care outcomes for individuals suffering with cancer-associated wasting [12,14].

Cachexia significantly affects health outcomes and effectiveness of treatments in cancer patients, greatly reducing the quality of life. It results in muscle wasting, persistent fatigue, and loss of physical function, which together hinder daily activities and overall well-being. The syndrome is also associated with psychological distress, such as anxiety and depression, adding to the patient’s burden [15,16]. Furthermore, cachexia is a strong predictor of poor prognosis and shortened survival in cancer patients; thus, early detection and care are essential for improving both clinical results and quality of life.

## Cancer cachexia and muscle loss

Muscle undergoes a drastic reduction in mass during the progression of cancer cachexia that, on the other hand, modulates whole-body metabolic homeostasis. It is caused by complex interplay between tumor-derived factors, patient’s metabolic dysregulation, psychological stressors, and anorexia-induced fat loss [17–19]. Patients usually lose more than 5% of their body weight, which is mainly due to loss of muscle along with reduction of body fat. Tumors act like “energy thieves,” reprogramming host’s systemic energy metabolism and leaving less energy for maintenance activity of the skeletal muscle. At the same time, chronic inflammation, indicated by elevated inflammatory cytokines, further worsens muscle health and accelerates tissue loss [20,21]. The illness is further exacerbated by other symptoms in the patient like anorexia, anemia, asthenia, pain, and anxiety [22]. Cachexia prevalence ranges from 50% to 90% in cancer patients, particularly those with pancreatic cancer, gastric cancer, and lung cancers. Muscle loss is also augmented by cancer treatment methods like chemotherapy and radiotherapy [23]. These treatments induce cachexia by disrupting protein homeostasis, specifically suppressing AKT/mTOR-mediated anabolic signaling and enhancing proteolytic pathways such as the ubiquitin-proteasome system (UPS) [24–26]. Chemotherapeutic agents enhance muscle cell apoptosis and cause energy deficits by affecting oxidative phosphorylation and mitochondrial dynamics [27]. Additionally, the senescence-associated secretory phenotype (SASP) promotes muscle degeneration by enhancing chronic inflammation through increased cytokine production. NF-κB signaling, together with C/EBPβ, drives the SASP by activating key cytokines such as IL-1α, IL-6, and IL-8 [28]. Muscle fiber loss and weakening are caused by neuromuscular dysfunction, which also emerges during cancer progression and treatment. This dysfunction is characterized by a decrease in motor unit number estimation (MUNE) and compound muscle action potential (CMAP) [29,30]. Furthermore, cancer-induced anabolic resistance and impairments in basal protein synthesis disrupt normal muscle regeneration and repair, thereby shifting the metabolic balance toward catabolism. Emerging evidence from murine models and clinical studies identifies the metal-ion transporter ZRT/IRT-like protein 14 (ZIP14) as a key mediator of cancer-associated muscle wasting. ZIP14 is up-regulated in cachectic skeletal muscle in response to pro-inflammatory cytokines such as TNF-α and TGF-β, leading to increased zinc influx. This disrupts myogenic regulators (MyoD, MEF2C), impairing muscle progenitor cell differentiation and promoting degradation of myosin heavy chain in mature muscle fibers [31]. Interestingly, recent studies show that adipose tissue acts as a key driver of cancer-associated muscle loss through dysregulated adipokine secretion, enhanced lipolysis, and browning of white adipose tissue (WAT) [32,33]. This adipose tissue and skeletal muscle cross-talk promotes energetically inefficient expenditure and accelerates muscle atrophy, ultimately leading to impaired physical function, reduced tolerance to anticancer therapies, and adverse clinical outcomes. These observations underscore the urgent need for targeted therapeutic strategies aimed at preserving skeletal muscle mass in cancer patients using more holistic approaches.

## Pro-inflammatory cytokines—central mediators of cancer cachexia

**Figure 1:**
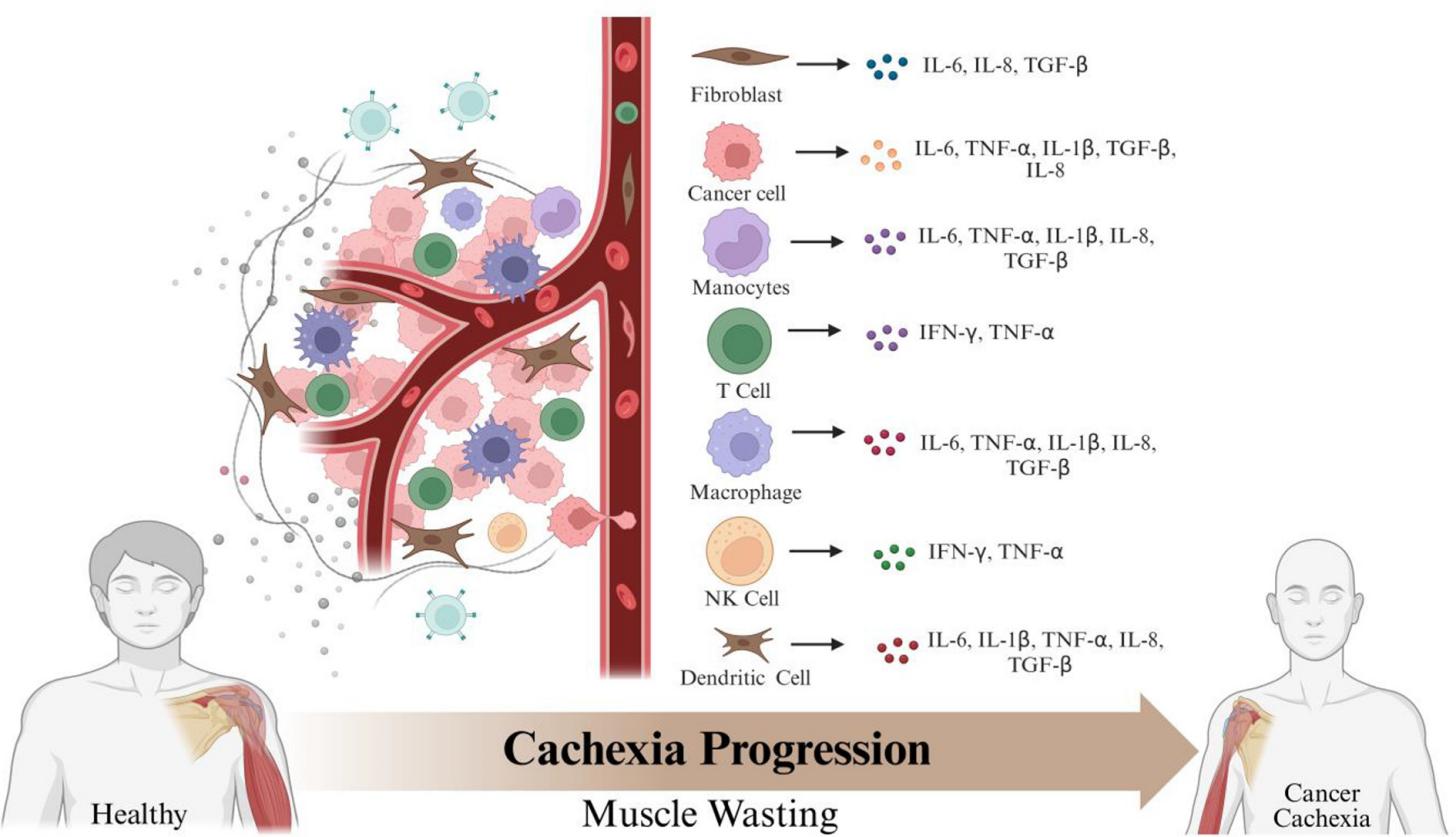
Inflammation-driven muscle loss in cancer cachexia Tumor microenvironment contains several types of cells apart from cancer cells, such as immune cells. Various different pro-inflammatory cytokines are produced by these cell types that modulate muscle metabolic processes.

Recent studies have indicated that a central driver of cancer cachexia pathogenesis is chronic systemic inflammation, where levels of circulatory pro-inflammatory cytokines remain elevated [34]. These signaling molecules exert profound effects on muscle metabolism by modulating inflammatory responses, energy homeostasis, and the processes governing muscle growth, regeneration, and repair [35,36]. Acting as molecular communicators between immune cells and skeletal muscle, these cytokines coordinate muscle wasting along with anorexia and global metabolic dysfunction in cancer patients [1,37]. Interestingly, pro-inflammatory cytokines are released in response to tumorigenesis, tissue damage, or infection by diverse immune cell populations, including macrophages, T lymphocytes, and NK cells, influencing the cachectic state (Figure 1). Once secreted, these low-molecular-weight mediators activate complex intracellular signaling networks that regulate inflammation, metabolic flux, and immune cell function. While they play essential roles in antitumor immunity, their sustained overproduction disrupts metabolic equilibrium, accelerates protein and lipid catabolism, and suppresses regenerative pathways [38,39]. Consequently, pro-inflammatory cytokines have emerged as critical and highly promising therapeutic targets for cancer cachexia. However, the complexity and extensive cross-talk among these signaling networks underscore the urgent need for a deeper and more integrated understanding to enable the rational design of precise interventions for cancer cachexia.

### Interleukin-6

Interleukin-6 (IL-6) (which can be secreted by immune cells and adipocytes) has emerged as a central mediator of metabolic dysfunction in cancer cachexia in both preclinical models and clinical studies of pancreatic, and colorectal cancers [40–42]. Persistent IL-6 exposure in *in vitro* studies using C2C12 myotubes and *in vivo* murine cachexia models induces anabolic resistance by impairing insulin–Akt–mTOR signaling, thereby limiting regeneration and repair capacity of muscle [43,44]. In adipose tissue, IL-6 promotes lipolysis and white adipose tissue browning through up-regulation of HSL, ATGL, and thermogenic genes such as UCP1, as demonstrated in rodent tumor-bearing models [45,46]. This remodeling increases energy expenditure and accelerates fat loss, worsening the negative energy balance characteristic of cachexia. Evidence from both experimental (animal models) and clinical studies identifies IL-6 as a key mediator linking skeletal muscle wasting with adipose dysfunction, making anti–IL-6 therapies such as siltuximab and tocilizumab promising interventions [47].

### Interferon-gamma

Interferon-gamma (IFN-γ) is strongly associated with cancer cachexia, affecting both skeletal muscle and adipose tissue, particularly in preclinical models of melanoma and lung cancer [48,49]. In skeletal muscle, sustained IFN-γ activates JAK–STAT1 signaling as demonstrated in *in vitro* and murine models, suppressing myogenic differentiation, impairing satellite cell function, and promoting protein degradation through UPS and autophagy–lysosomal pathways [50,51]. IFN-γ also synergizes with TNF-α and IL-6 to exacerbate muscle wasting by inhibiting Akt–mTOR–mediated protein synthesis and increasing oxidative stress [52,53]. In adipose tissue, it disrupts lipid homeostasis by inhibiting adipocyte differentiation, reducing lipid storage, and promoting inflammatory remodeling and macrophage infiltration, thereby stimulating lipolysis in human adipocyte studies [54,55]. Genetic deletion or blockade of IFN-γ has been shown to attenuate muscle and fat loss in murine models [56,57].

### Tumor necrosis factor-alpha

Tumor necrosis factor-alpha (TNF-α), historically known as cachectin, is one of the earliest cytokines implicated in cancer cachexia based on both clinical observations and experimental tumor models such as pancreatic and gastric cancers [58–61]. Interestingly, TNF-α exerts pathological effects on both skeletal muscle and adipose tissue. In skeletal muscle, TNF-α activates NF-κB and p38 MAPK signaling in *in vitro* myoblast studies [62], up-regulating MuRF1 and Atrogin-1 and accelerating proteolysis via the UPS while suppressing myogenic differentiation, hence regeneration capacity [63]. TNF-α also interferes with insulin–Akt–mTOR signaling, thereby reducing protein synthesis and promoting anabolic resistance [64]. In adipose tissue, TNF-α stimulates lipolysis through HSL and ATGL and inhibits adipogenesis by down-regulating PPARγ and C/EBPα, leading to fat loss and metabolic imbalance in rodent models [65,66]. Inhibition of TNF-α using agents such as infliximab, adalimumab, and etanercept attenuates cachectic features [67].

### Transforming growth factor-beta

Experimental evidence implicates transforming growth factor-beta (TGF-β) as a key cytokine in the progression of cancer cachexia, affecting both skeletal muscle and adipose tissue in pancreatic, colorectal, and lung cancers [68,69]. In skeletal muscle, elevated TGF-β signaling through SMAD3 has been demonstrated in *in vitro* and *in vivo* studies, suppresses myogenic differentiation by inhibiting MyoD and myogenin, impairs satellite cell–mediated regeneration, and promotes muscle fibrosis, contributing to muscle atrophy [70]. Members of the TGF-β family, including myostatin and activins, further exacerbate wasting by reducing Akt–mTOR–dependent protein synthesis and enhancing proteolysis [71,72]. In adipose tissue, TGF-β inhibits adipogenesis by repressing PPARγ and C/EBPα, limiting lipid storage and promoting adipose remodeling in rodent models [73,74]. Antibody-mediated inhibition using fresolimumab attenuates cachectic features in rodent models, although clinical validation remains necessary [75–77].

### Interleukin-1 beta

Interleukin-1 beta (IL-1β) is also involved in cancer cachexia through its effects on both skeletal muscle and adipose tissue, as evidenced by *in vitro* and *in vivo* models, and selected clinical observations in cancer patients. In skeletal muscle, IL-1β activates NF-κB and p38 MAPK signaling, in C2C12 myotubes and tumor-bearing mice [78,79], increasing expression of muscle-specific ubiquitin ligases and promoting protein degradation via UPS and autophagy–lysosomal pathways [80,81]. Persistent IL-1β signaling also impairs insulin and Akt–mTOR pathways, contributing to anabolic resistance and reduced muscle regeneration [82]. In adipose tissue, IL-1β stimulates lipolysis, enhances macrophage infiltration, and inhibits adipocyte differentiation through down-regulation of PPARγ in rodent models [83,84]. These changes accelerate fat loss and metabolic imbalance. Neutralization of IL-1β using canakinumab has shown clinical potential in reducing inflammation and improving energy balance [81].

### Interleukin-8

Interleukin-8 (IL-8) (also known as CXCL8) is a chemokine that modulates metabolism in skeletal muscle and adipose tissue potentially implicated in cancer cachexia based on evidence from *in vitro* studies, *in vivo* tumor-bearing models, and limited clinical observations in patients with solid tumors such as lung and colorectal cancers [85]. In skeletal muscle, elevated IL-8 activates CXCR1/CXCR2 signaling in cultured myotubes and murine cancer cachexia models, increasing oxidative stress, impairing myogenic differentiation, and enhancing proteolytic activity that contributes to muscle wasting [86,87]. IL-8 is also associated with reduced insulin sensitivity and mitochondrial dysfunction in muscle fibers, further aggravating metabolic inefficiency [88]. In adipose tissue, IL-8 promotes inflammatory remodeling by recruiting immune cells and stimulating lipolysis, thereby accelerating fat loss and disrupting adipocyte metabolic stability in rodent models [89–91]. Although antibody-based inhibition of IL-8 (e.g., BMS-986253) is feasible, therapeutic benefits remain limited, probably due to chemokine redundancy [92].

## Inflammatory signaling underlying skeletal muscle wasting in cancer cachexia

**Figure 2:**
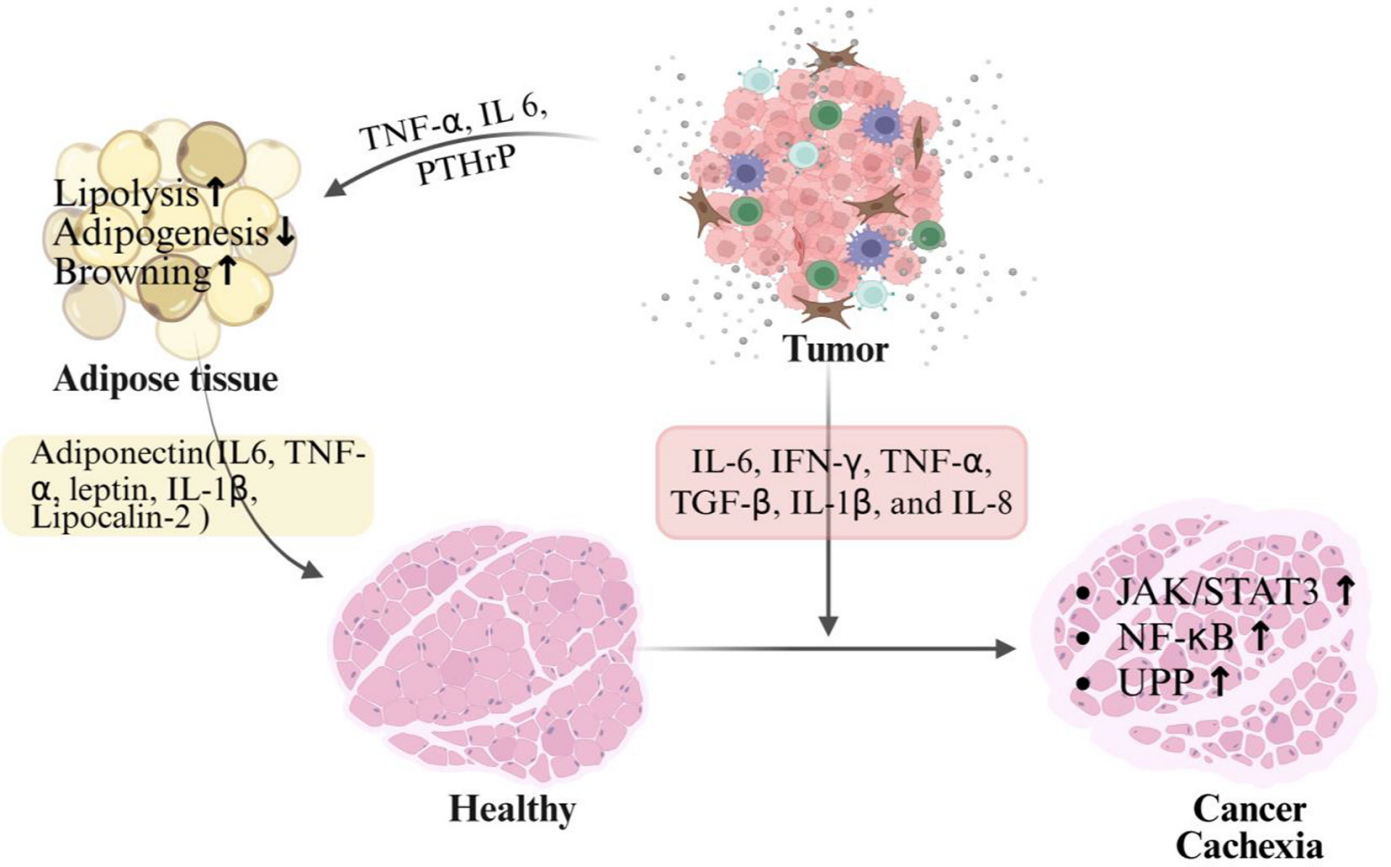
Viscous cycle of inflammation: tumor–adipose tissue–muscle Tumor-secreted factors induce adipose tissue remodeling, including beiging and browning. Structural and functional remodeling of adipose tissue leads to altered adipokine secretion, which in turn amplifies catabolic and cachectic signaling pathways in skeletal muscle.

Chronic exposure to pro-inflammatory cytokines drives profound bioenergetic inefficiency in skeletal muscle by disrupting substrate utilization. Inflammatory signaling impairs oxidative phosphorylation, reduces mitochondrial ATP-generating capacity, and enhances reactive oxygen species production, disturbing metabolic efficiency [93]. Concurrently, cytokines promote metabolic inflexibility by blunting insulin-stimulated glucose uptake and shifting muscle substrate preference toward lipid and amino acid oxidation. It is interesting to note that these cytokines originate not only from tumor cells but also from infiltrating immune cells and adipose tissue, creating a systemic inflammatory environment that targets skeletal muscle fibers. These signals activate catabolic pathways in muscle, particularly the JAK-STAT signaling pathway and NF-κB signaling pathway, which enhance protein degradation through the UPS pathway. The resulting inflammatory and metabolic interactions between tumor and adipose tissue, drives tissue wasting and metabolic imbalance of the skeletal muscle during cancer cachexia (Figure 2).

### NF-κB signaling pathway

The NF-κB pathway serves as a central modulator of immune and inflammatory responses and plays a pivotal role in the onset and progression of cancer cachexia [94]. It drives the transcription in response to pro-inflammatory cytokines such as TNF-α, IL-6, IL-1β, and IFN-γ, as well as chemokines like MCP-1 [95]. NF-κB is activated downstream of receptors including TNFR1, IL-1R1, and Toll-like receptors (e.g., TLR4) by binding of the above cytokines [96]. Upon activation, NF-κB translocates to the nucleus and up-regulates several genes that promote inflammation and muscle catabolism [97,98]. Key NF-κB-mediated effects include increased expression of muscle-specific E3 ubiquitin ligases such as atrogin-1 and muscle RING-finger protein-1 (MuRF1). MuRF1 plays a central role in myofibrillar protein turnover by targeting major contractile proteins, including myosin heavy chain, for ubiquitination and proteasomal degradation [99,100]. Moreover, NF-κB impairs protein synthesis by down-regulating the AKT/mTOR pathway and contributes to mitochondrial dysfunction and disruption of energy homeostasis in cachectic patients.

### JAK/STAT3 signaling pathway

The JAK/STAT3 pathway is another major signaling axis contributing to cachexia, especially through the actions of IL-6 and related cytokines. Cytokine binding triggers JAK activation, which in turn phosphorylates STAT3, enabling its dimerization and nuclear translocation, where it regulates transcription of genes involved in metabolic dysfunction. In skeletal muscle, STAT3 activation suppresses protein synthesis by inhibiting the AKT/mTOR axis and promotes protein degradation by up-regulating both the UPS and the autophagy–lysosome pathway. In addition to IL-6, IFN-γ can also activate JAK/STAT3 signaling in skeletal muscle, further amplifying inflammatory and catabolic responses. Multiple JAK isoforms, including JAK1, JAK2, and TYK2, are expressed in skeletal muscle, allowing diverse cytokine signals to converge on STAT3 activation during cachexia [101,102]. Systemically, sustained activation of STAT3 signaling contributes to the metabolic derangements of cancer cachexia by reprogramming peripheral organ function; for example, in liver, it activates gluconeogenic gene expression (e.g., PEPCK, G6PC, and PGC-1α) [103]. In adipose tissue, STAT3 signaling promotes lipolysis through the induction of lipolytic enzymes (ATGL and HSL) and inflammatory mediators (IL-6 and TNF-α), leading to accelerated fat loss [104]. Together, these effects intensify whole-body energy imbalance, amplify substrate depletion, and aggravate the muscle catabolism that underlies the progression of cachexia.

### Ubiquitin-proteasome pathway

The UPS pathway represents the primary system for intracellular protein degradation and is markedly up-regulated during cancer-associated muscle wasting. The process involves the sequential action of three classes of enzymes: E1 (activating), E2 (conjugating), and E3 (ligating), which coordinate the tagging of target proteins with ubiquitin [105]. Muscle-specific E3 ligases, especially atrogin-1 (also called MAFbx) and MuRF1, are selectively induced under catabolic stress and facilitate the proteasomal degradation of essential myofibrillar proteins, culminating in muscle loss [106]. Pro-inflammatory cytokines such as TNF-α, IL-6, and IL-1β promote skeletal muscle wasting in cancer cachexia by activating NF-κB and FOXO signaling, leading to increased expression of the muscle-specific E3 ligases MuRF1 and Atrogin-1 and subsequent UPS hyperactivation. This pathway has been therapeutically targeted mainly at the cytokine level; for example, TNF-α antagonists and IL-6 blockade have been shown to attenuate muscle proteolysis and preserve muscle mass in preclinical models and few clinical studies. In contrast, direct inhibition of the E3 ligases, although effective experimentally, has limited clinical applicability due to toxicity and disruption of normal protein turnover [107]. These findings establish cytokine-driven UPS activation as a central and targetable mechanism in cancer cachexia.

### Adipose tissue as a pro-inflammatory amplifier in cancer cachexia

Over the past decade, WAT has emerged as a dynamic endocrine organ capable of amplifying inflammatory responses in several metabolic disorders such as obesity and nonalcoholic fatty liver disease. In the context of cancer cachexia, WAT undergoes profound structural and functional remodeling driven by tumor progression. Tumor cells release multiple pro-inflammatory mediators, including IL-6, IFN-γ, TNF-α, and TGF-β, which initiate metabolic reprogramming of adipose tissue. These factors promote enhanced lipolysis, adipocyte shrinkage, and the browning of WAT, thereby increasing thermogenic activity and systemic energy expenditure. As adipose tissue remodels, it becomes an important source of adipokines and inflammatory mediators, such as leptin, adiponectin, IL-1β, and lipocalin-2, which further intensify systemic inflammation. This inflammatory adipose environment strongly influences skeletal muscle by activating catabolic pathways, including the JAK-STAT, NF-κB, and UPS pathways. Consequently, adipose tissue acts as a critical pro-inflammatory amplifier that strengthens tumor-induced metabolic disturbances, establishing a vicious tumor–adipose–muscle cycle that accelerates tissue wasting during cancer cachexia (Figure 2).

## Clinical implications

The growing understanding of cytokine-driven signaling networks has begun to ignite therapeutic strategies aimed at interrupting the inflammatory drivers of cachexia (Table 1). Targeting the IL-6–STAT3 axis has emerged as a particularly promising approach, given its central role in sustaining systemic inflammation and muscle catabolism. Pharmacological inhibition using monoclonal antibodies against IL-6 or IL-6 receptor, such as tocilizumab, has shown encouraging results in preclinical and early clinical studies by reducing muscle degradation in murine tumor models [108]. Similarly, blockade of TNF-α using agents such as infliximab, etanercept, and thalidomide has been explored to dampen NF-κB activation and limit inflammation-induced proteolysis, retarding cancer cachexia in preclinical studies [109,110]. Beyond classical inflammatory cytokines, emerging mediators such as growth differentiation factor-15 (GDF-15, a TGF-β agonist) have gained attention due to their strong association with anorexia and metabolic dysregulation in cachexia. Neutralization of GDF-15 signaling has been shown to restore appetite, increase physical activity, and indirectly mitigate muscle loss in experimental animal models, highlighting the importance of targeting both metabolic and inflammatory pathways [111]. In parallel, pharmacological modulation of JAK/STAT signaling represents another promising therapeutic avenue for disrupting cytokine-induced catabolic signaling in skeletal muscle.

**Table 1:** Cytokine-targeted therapeutic strategies in cancer cachexia

Cytokine	Therapeutic agent	Study type
IL-6	Tocilizumab	Clinical + Preclinical
IL-6	Siltuximab	Clinical
TNF-α	Infliximab	Clinical
TNF-α	Etanercept	Clinical + Preclinical
TNF-α	Thalidomide	Clinical
IFN-γ	Neutralizing antibodies	Preclinical
TGF-β	Fresolimumab	Preclinical
IL-1β	Canakinumab	Clinical
IL-8	BMS-986253	Clinical
GDF-15	Anti–GDF-15 antibodies	Preclinical

However, clinical experience indicates that targeting a single cytokine pathway often yields limited benefits due to the redundancy and interconnectedness of inflammatory networks. Consequently, current therapeutic strategies increasingly emphasize multimodal approaches that combine anti-inflammatory interventions with metabolic and anabolic support. Agents such as megestrol acetate and dronabinol are used to stimulate appetite, while anabolic therapies including oxandrolone or nandrolone decanoate aim to preserve muscle mass [112–114]. Nutritional supplementation with omega-3 fatty acids and structured exercise programs further contribute to maintaining metabolic homeostasis. Together, these strategies underscore the need for integrated therapeutic regimens that address both inflammation and metabolic dysfunction in cancer cachexia. Importantly, circulating cytokines and their downstream signaling components are also emerging as valuable biomarkers for disease progression and therapeutic response [115]. Monitoring cytokine signatures may enable earlier identification of patients at risk of cachexia and facilitate personalized intervention strategies. Thus, translating mechanistic insights into clinically actionable biomarkers and targeted therapies represents a critical step toward improving the management of cancer cachexia.

## Future perspectives

Despite substantial advances in understanding the molecular drivers of cancer cachexia, effective clinical therapies remain limited. One of the key challenges lies in the complex and redundant cytokine networks that sustain systemic inflammation and metabolic dysregulation. Future research must therefore move beyond the study of individual cytokines toward a systems-level understanding of the inflammatory circuits that integrate tumor signals with host metabolic responses [116]. In particular, deciphering how cytokine signaling interfaces with major catabolic pathways such as NF-κB, JAK/STAT3, and the UPS will be essential for identifying nodal regulatory points that can be therapeutically targeted [117]. Another critical area of investigation that needs to be addressed is the role of inter-organ communication in cachexia. Increasing evidence indicates that cancer cachexia is not solely a muscle-centric disorder (and probably does not originate in the muscle as well) but rather a multi-organ syndrome involving coordinated interactions among various systems, including skeletal muscle, adipose tissue, liver, and immune cells. Cytokines derived from tumors and immune cells influence adipose tissue lipolysis, hepatic acute-phase responses, and hypothalamic appetite regulation, creating a vicious cycle of inflammation and metabolic imbalance. Understanding this tumor–immune–muscle–adipose axis may reveal new therapeutically targetable points capable of disrupting the systemic propagation of cancer cachexia.

Emerging molecular pathways also offer promising opportunities for therapeutic development. For instance, TGF-β family signaling has been implicated in suppressing muscle regeneration and promoting fibrosis, suggesting that modulation of this pathway could restore muscle plasticity in cachectic patients [68]. Similarly, recent studies indicate that inflammatory cytokines can promote adipose tissue browning and increased energy expenditure, further exacerbating weight loss [118]. Targeting the molecular mechanisms that regulate adipose remodeling and energy metabolism may therefore complement strategies aimed at preserving muscle mass. Technological advances in molecular research in last few decades are expected to play a pivotal role in accelerating these discoveries. High-resolution approaches such as single-cell transcriptomics, spatial omics, and proteomic profiling can provide unprecedented insights into cell-specific cytokine signaling and the dynamic interactions between tumor and host tissues. These tools may facilitate the identification of novel biomarkers and enable the stratification of patients according to inflammatory signatures, thereby improving the design of precision therapies. Ultimately, the future management of cancer cachexia will likely rely on integrated therapeutic strategies that combine cytokine modulation with metabolic and regenerative interventions. Such approaches may include targeted inhibition of key inflammatory pathways, pharmacological stimulation of anabolic signaling, nutritional optimization, and exercise-based rehabilitation programs. By bridging mechanistic insights with translational research, these multidisciplinary strategies hold significant promise for transforming the clinical management of cancer cachexia patients.

Importantly, evidence suggests that biological sex may significantly influence the progression of cancer cachexia and cytokine signaling responses. Sex-specific differences in immune function, hormonal regulation, and muscle metabolism can modulate the activity of pro-inflammatory cytokines such as IL-6 and TNF-α. For instance, estrogen has been shown to exert anti-inflammatory effects and may partially protect against excessive muscle wasting, whereas testosterone influences anabolic signaling pathways in skeletal muscle [119,120]. More broadly, males and females exhibit fundamental differences in immune responses, with females generally showing heightened immune reactivity, which may contribute to their increased susceptibility to autoimmune and inflammatory disorders, particularly during the reproductive phase. These differences are driven in part by sex hormones, which distinctly regulate immune cell function and cytokine production. In addition, sex-specific metabolic characteristics, where females tend to favor adipose tissue storage and males exhibit greater skeletal muscle mass, may further influence the severity and progression of cachexia. Despite these well-established biological differences, most preclinical and clinical studies have not systematically incorporated sex as a critical variable. Therefore, integrating sex-based analyses is essential to better understand differential disease susceptibility and to develop more precise and effective therapeutic strategies for cancer cachexia.

## Perspectives

- **Highlight the importance of the field-** Cancer cachexia is highly prevalent more than 50% among patients of solid tumors such as. Recent therapies targeting inflammatory signaling pathways showing control of cancer cachexia suggest inflammatory pathways as a major mechanism in the pathogenesis of muscle loss.
- **Summary of the current thinking-** Tumor-derived pro-inflammatory cytokines such as IL-6, TNF-α, IFN-γ, and TGF-β activate catabolic signaling pathways accelerating skeletal muscle wasting. These cytokines also influence adipose tissue to modulate secretion of various adipokines having potential to affect muscle metabolism.
- **Comment on future directions-** Advances in multi-omics technologies and systems-level approaches are expected to reveal novel biomarkers and therapeutic targets within the tumor–adipose–muscle network, enabling more precise and effective treatment strategies for cancer cachexia.

## Open Access

Open access for this article is provided via our Subscribe to Open programme, made possible through the support of our subscribing institutions. Learn more here: https://portlandpress.com/pages/open_access_options_and_prices#conditional.

## Abbreviation

ATGL: Adipose triglyceride lipase
CMAP: Compound muscle action potential
HSL: Hormone-sensitive lipase
IFN-γ: Interferon-gamma
IGF-1: Insulin-like growth factor-1
IL-1β: Interleukin-1 beta
IL-6: Interleukin-6
IL-8: Interleukin-8
MUNE: Motor unit number estimation
NF-κB: Nuclear Factor-κB
NK: Natural Killer
SASP: Senescence-associated secretory phenotype
TGF-β: Transforming growth factor-beta
TNF-α: Tumor necrosis factor-α
UCP1: Uncoupling protein
UPS: ubiquitin-proteasome system

## References

[1] Webster J.M., Kempen L., Hardy R.S. and Langen R.C.J. (2020) Inflammation and skeletal muscle wasting during cachexia. Front Physiol. 11, 597675 10.3389/fphys.2020.597675 33329046 PMC7710765

[2] Fearon K., Strasser F., Anker S.D., Bosaeus I., Bruera E., Fainsinger R.L. et al. (2011) Definition and classification of cancer cachexia: an international consensus. Lancet Oncol. 12, 489–495 10.1016/S1470-2045(10)70218-7 21296615

[3] Thanapholsart J., Khan E., Ismail T.F. and Lee G.A. (2023) The complex pathophysiology of cardiac cachexia: a review of current pathophysiology and implications for clinical practice. Am. J. Med. Sci. 365, 9–18 10.1016/j.amjms.2022.08.016 36055378

[4] Schols A.M. (2002) Pulmonary cachexia. Int. J. Cardiol. 85, 101–110 10.1016/S0167-5273(02)00238-3 12163214

[5] Okamura M., Konishi M., Butler J., Kalantar-Zadeh K., von Haehling S. and Anker S.D. (2023) Kidney function in cachexia and sarcopenia: facts and numbers. J. Cachexia Sarcopenia Muscle 14, 1589–1595 10.1002/jcsm.13260 37222019 PMC10401526

[6] Bellelli A., Santi D., Simoni M. and Greco C. (2022) Diabetic neuropathic cachexia: a clinical case and review of literature. Life (Basel) 12, 680 10.3390/life12050680 35629348 PMC9147421

[7] Von Roenn J.H., Roth E.L. and Craig R. (1992) HIV-related cachexia: potential mechanisms and treatment. Oncology 49, 50–54 1461629 10.1159/000227129

[8] Martin L., Muscaritoli M., Bourdel-Marchasson I., Kubrak C., Laird B., Gagnon B. et al. (2021) Diagnostic criteria for cancer cachexia: reduced food intake and inflammation predict weight loss and survival in an international, multi-cohort analysis. J. Cachexia Sarcopenia Muscle 12, 1189–1202 10.1002/jcsm.12756 34448539 PMC8517347

[9] Malla J., Zahra A., Venugopal S., Selvamani T.Y., Shoukrie S.I., Selvaraj R. et al. (2022) What role do inflammatory cytokines play in cancer cachexia? Cureus 14, e26798 10.7759/cureus.26798 35971351 PMC9372379

[10] Rohm M., Zeigerer A., Machado J. and Herzig S. (2019) Energy metabolism in cachexia. EMBO Rep. 20, e47258 10.15252/embr.201847258 30890538 PMC6446208

[11] Meza-Valderrama D., Marco E., Dávalos-Yerovi V., Muns M.D., Tejero-Sánchez M., Duarte E. et al. (2021) Sarcopenia, malnutrition, and cachexia: adapting definitions and terminology of nutritional disorders in older people with cancer. Nutrients 13, 761 10.3390/nu13030761 33652812 PMC7996854

[12] Ni J. and Zhang L. (2020) Cancer cachexia: definition, staging, and emerging treatments. Cancer Manag. Res. 12, 5597–5605 10.2147/CMAR.S261585 32753972 PMC7358070

[13] Rogers J.B., Syed K. and Minteer J.F. (2023) Cachexia. StatPearls, StatPearls Publishing 29262118

[14] Vaughan V.C., Martin P. and Lewandowski P.A. (2013) Cancer cachexia: impact, mechanisms and emerging treatments. J. Cachexia Sarcopenia Muscle 4, 95–109 10.1007/s13539-012-0087-1 23097000 PMC3684701

[15] Baker Rogers J., Syed K. and Minteer J.F. (2025) Cachexia. StatPearls, StatPearls Publishing, Treasure Island (FL), Copyright © 2025, StatPearls Publishing LLC. 29262118

[16] Sun H., Sudip T., Fu X., Wen S., Liu H. and Yu S. (2023) Cachexia is associated with depression, anxiety and quality of life in cancer patients. BMJ Support Palliat Care 13, e129–e135 10.1136/bmjspcare-2019-002176 32917649

[17] Yue M., Qin Z., Hu L. and Ji H. (2024) Understanding cachexia and its impact on lung cancer and beyond. Chin. Med. J. Pulm. Crit. Care Med. 2, 95–105 10.1016/j.pccm.2024.02.003 39169934 PMC11332896

[18] Gilmore L.A., Parry T.L., Thomas G.A. and Khamoui A.V. (2023) Skeletal muscle omics signatures in cancer cachexia: perspectives and opportunities. JNCI Monographs 2023, 30–42 10.1093/jncimonographs/lgad006 PMC10157770 37139970

[19] Hopkinson J.B. (2014) Psychosocial impact of cancer cachexia. J. Cachexia Sarcopenia Muscle 5, 89–94 10.1007/s13539-014-0142-1 24737110 PMC4053560

[20] Tufail M., Jiang C.H. and Li N. (2024) Altered metabolism in cancer: insights into energy pathways and therapeutic targets. Mol. Cancer 23, 203 10.1186/s12943-024-02119-3 39294640 PMC11409553

[21] Jaffer U., Wade R.G. and Gourlay T. (2010) Cytokines in the systemic inflammatory response syndrome: a review. HSR Proc. Intensive Care Cardiovasc. Anesth. 2, 161–175 23441054 PMC3484588

[22] Muthanandam S. and Muthu J. (2021) Understanding cachexia in head and neck cancer. Asia-Pacific J. Oncol. Nursing 8, 527–538 10.4103/apjon.apjon-2145 PMC8420913 34527782

[23] Coletti D. (2018) Chemotherapy-induced muscle wasting: an update. Eur. J. Transl. Myol. 28, 7587 10.4081/ejtm.2018.7587 29991991 PMC6036312

[24] Aversa Z., Costelli P. and Muscaritoli M. (2017) Cancer-induced muscle wasting: latest findings in prevention and treatment. Ther. Adv. Med. Oncol. 9, 369–382 10.1177/1758834017698643 28529552 PMC5424865

[25] Meric-Bernstam F. and Gonzalez-Angulo A.M. (2009) Targeting the mTOR signaling network for cancer therapy. J. Clin. Oncol. 27, 2278–2287 10.1200/JCO.2008.20.0766 19332717 PMC2738634

[26] LaPlante G. and Zhang W. (2021) Targeting the ubiquitin-proteasome system for cancer therapeutics by small-molecule inhibitors. Cancers (Basel) 13, 3079 10.3390/cancers13123079 34203106 PMC8235664

[27] Gorini S., De Angelis A., Berrino L., Malara N., Rosano G. and Ferraro E. (2018) Chemotherapeutic drugs and mitochondrial dysfunction: focus on doxorubicin, trastuzumab, and sunitinib. Oxid. Med. Cell Longev. 2018, 7582730 10.1155/2018/7582730 29743983 PMC5878876

[28] Zhao S., Qiao Z., Pfeifer R., Pape H.C., Mao K., Tang H. et al. (2024) Modulation of fracture healing by senescence-associated secretory phenotype (SASP): a narrative review of the current literature. Eur. J. Med. Res. 29, 38 10.1186/s40001-023-01604-7 38195489 PMC10775505

[29] Huot J.R., Pin F. and Bonetto A. (2021) Muscle weakness caused by cancer and chemotherapy is associated with loss of motor unit connectivity. Am. J. Cancer Res. 11, 2990–3001 34249440 PMC8263661

[30] Rashidipour O. and Chan K.M. (2008) Motor unit number estimation in neuromuscular disease. Can. J. Neurol. Sci. 35, 153–159 10.1017/S0317167100008568 18574927

[31] Wang G., Biswas A.K., Ma W., Kandpal M., Coker C., Grandgenett P.M. et al. (2018) Metastatic cancers promote cachexia through ZIP14 upregulation in skeletal muscle. Nat. Med. 24, 770–781 10.1038/s41591-018-0054-2 29875463 PMC6015555

[32] Vaitkus J.A. and Celi F.S. (2017) The role of adipose tissue in cancer-associated cachexia. Exp. Biol. Med. (Maywood) 242, 473–481 10.1177/1535370216683282 27932592 PMC5367652

[33] Zhang X., Han X., Xu J. and Li G. (2026) Disease-associated adipose browning: current evidence and perspectives. Adipocyte 15, 2610540 10.1080/21623945.2025.2610540 41498391 PMC12785212

[34] Tan Y., Xue R., Pan Y., He Z., Hu X., Li Y. et al. (2026) Cancer cachexia: molecular basis and therapeutic advances. Signal Transduct. Targeted Therapy 11, 16 10.1038/s41392-025-02331-7 PMC12795897 41526343

[35] Sharma B. and Dabur R. (2020) Role of pro-inflammatory cytokines in regulation of skeletal muscle metabolism: a systematic review. Curr. Med. Chem. 27, 2161–2188 10.2174/0929867326666181129095309 30488792

[36] Tan Y., Xue R., Pan Y., He Z., Hu X., Li Y. et al. (2026) Cancer cachexia: molecular basis and therapeutic advances. Signal Transduct. Target Ther. 11, 16 10.1038/s41392-025-02331-7 41526343 PMC12795897

[37] Bilski J., Szlachcic A., Ptak-Belowska A. and Brzozowski T. (2025) Physical activity, exerkines, and their role in cancer cachexia. Int. J. Mol. Sci. 26, 8011 10.3390/ijms26168011 40869331 PMC12386252

[38] Kany S., Vollrath J.T. and Relja B. (2019) Cytokines in inflammatory disease. Int. J. Mol. Sci. 20, 6008 10.3390/ijms20236008 31795299 PMC6929211

[39] Tahmasebi S., Alimohammadi M., Khorasani S. and Rezaei N. (2025) Pro-tumorigenic and anti-tumorigenic roles of pro-inflammatory cytokines in cancer. In Cancer Immunology: The Immune System and Tumor(Rezaei N., ed.), pp. 529–553, Springer Nature Switzerland, Cham 10.1007/978-3-032-00749-0_25

[40] Agca S. and Kir S. (2024) The role of interleukin-6 family cytokines in cancer cachexia. FEBS J. 291, 4009–4023 10.1111/febs.17224 38975832

[41] White J.P. (2017) IL-6, cancer and cachexia: metabolic dysfunction creates the perfect storm. Transl. Cancer Res. 6, S280–S285 10.21037/tcr.2017.03.52 30766805 PMC6372111

[42] Holmer R., Goumas F.A., Waetzig G.H., Rose-John S. and Kalthoff H. (2014) Interleukin-6: a villain in the drama of pancreatic cancer development and progression. Hepatobiliary & Pancreatic Dis. Int. 13, 371–380 10.1016/S1499-3872(14)60259-9 25100121

[43] Yuen D.Y., Dwyer R.M., Matthews V.B., Zhang L., Drew B.G., Neill B. et al. (2009) Interleukin-6 attenuates insulin-mediated increases in endothelial cell signaling but augments skeletal muscle insulin action via differential effects on tumor necrosis factor-alpha expression. Diabetes 58, 1086–1095 10.2337/db08-0775 19188427 PMC2671037

[44] Li L., Huang C., Pang J., Huang Y., Chen X. and Chen G. (2023) Advances in research on cell models for skeletal muscle atrophy. Biomed. Pharmacotherapy 167, 115517 10.1016/j.biopha.2023.115517 37738794

[45] Radványi Á. and Röszer T. (2024) Interleukin-6: An under-appreciated inducer of thermogenic adipocyte differentiation. Int. J. Mol. Sci. 25, 2810 10.3390/ijms25052810 38474057 PMC10932467

[46] Wolsk E., Mygind H., Grøndahl T.S., Pedersen B.K. and van Hall G. (2010) IL-6 selectively stimulates fat metabolism in human skeletal muscle. Am. J. Physiol. Endocrinol. Metab. 299, E832–E840 10.1152/ajpendo.00328.2010 20823453

[47] Narsale A.A. and Carson J.A. (2014) Role of interleukin-6 in cachexia: therapeutic implications. Curr. Opin. Support Palliat Care 8, 321–327 10.1097/SPC.0000000000000091 25319274 PMC4323347

[48] Taniguchi K., Petersson M., Höglund P., Kiessling R., Klein G. and Kärre K. (1987) Interferon gamma induces lung colonization by intravenously inoculated B16 melanoma cells in parallel with enhanced expression of class I major histocompatibility complex antigens. Proc. Natl. Acad. Sci. U.S.A. 84, 3405–3409 10.1073/pnas.84.10.3405 3106968 PMC304879

[49] Kratzmeier C., Taheri M., Mei Z., Lim I., Khalil M.A., Carter-Cooper B. et al. (2025) Lung adenocarcinoma–derived IFN-γ promotes growth by modulating CD8+ T cell production of CCR5 chemokines. J. Clin. Invest. 135, e191070 10.1172/JCI191070 40553564 PMC12404755

[50] Hou C., Periou B., Gervais M., Martin L., Berthier J., Baba-Amer Y. et al. (2025) Interferon-γ causes myogenic cell dysfunction and senescence in immune myopathies. Brain 148, 2883–2898 10.1093/brain/awaf153 40296760

[51] Hou C., Periou B., Gervais M., Berthier J., Baba Amer Y., Malfatti E. et al. (2023) Interferon-gamma-mediated JAK/STAT1 signalling triggers muscle damage in Inclusion Body Myositis. Morphologie 107, 100625 10.1016/j.morpho.2023.100625

[52] Ma J.F., Sanchez B.J., Hall D.T., Tremblay A.K., Di Marco S. and Gallouzi I.E. (2017) STAT3 promotes IFNγ/TNFα-induced muscle wasting in an NF-κB-dependent and IL-6-independent manner. EMBO Mol. Med. 9, 622–637 10.15252/emmm.201607052 28264935 PMC5412921

[53] Mir M., Asensio V.J., Tolosa L., Gou-Fabregas M., Soler R.M., Lladó J. et al. (2009) Tumor necrosis factor alpha and interferon gamma cooperatively induce oxidative stress and motoneuron death in rat spinal cord embryonic explants. Neuroscience 162, 959–971 10.1016/j.neuroscience.2009.05.049 19477238

[54] McGillicuddy F.C., Chiquoine E.H., Hinkle C.C., Kim R.J., Shah R., Roche H.M. et al. (2009) Interferon gamma attenuates insulin signaling, lipid storage, and differentiation in human adipocytes via activation of the JAK/STAT pathway. J. Biol. Chem. 284, 31936–31944 10.1074/jbc.M109.061655 19776010 PMC2797265

[55] Yao J., Wu D. and Qiu Y. (2022) Adipose tissue macrophage in obesity-associated metabolic diseases. Front Immunol. 13, 977485 10.3389/fimmu.2022.977485 36119080 PMC9478335

[56] Matthys P., Dijkmans R., Proost P., Van Damme J., Heremans H., Sobis H. et al. (1991) Severe cachexia in mice inoculated with interferon-gamma-producing tumor cells. Int. J. Cancer 49, 77–82 10.1002/ijc.2910490115 1908442

[57] Mueller T.C., Bachmann J., Prokopchuk O., Friess H. and Martignoni M.E. (2016) Molecular pathways leading to loss of skeletal muscle mass in cancer cachexia – can findings from animal models be translated to humans? BMC Cancer 16, 75 10.1186/s12885-016-2121-8 26856534 PMC4746781

[58] Blay J.Y. and Chouaib S. (1989) Tumor necrosis factor alpha (cachectin). Biological properties and role in physiopathology. Presse Med. 18, 975–979 2525723

[59] Patel H.J. and Patel B.M. (2017) TNF-α and cancer cachexia: molecular insights and clinical implications. Life Sci. 170, 56–63 10.1016/j.lfs.2016.11.033 27919820

[60] Loyala J.V., Down B., Wong E. and Tan B. (2024) Treatment of cachexia in gastric cancer: exploring the use of anti-inflammatory natural products and their derivatives. Nutrients 16, 1246 10.3390/nu16081246 38674936 PMC11053965

[61] Xiao A., Feng Y., Yin B., Zhang J., Cao Z., Liu X. et al. (2025) Pancreatic cancer cachexia: a systemic consequence of multi-organ interactions. hLife 3, 576–614 10.1016/j.hlife.2025.05.002

[62] Chen S.E., Jin B. and Li Y.P. (2007) TNF-alpha regulates myogenesis and muscle regeneration by activating p38 MAPK. Am. J. Physiol. Cell Physiol. 292, C1660–C1671 10.1152/ajpcell.00486.2006 17151142 PMC3099536

[63] Egerman M.A. and Glass D.J. (2014) Signaling pathways controlling skeletal muscle mass. Crit. Rev. Biochem. Mol. Biol. 49, 59–68 10.3109/10409238.2013.857291 24237131 PMC3913083

[64] Plomgaard P., Bouzakri K., Krogh-Madsen R., Mittendorfer B., Zierath J.R. and Pedersen B.K. (2005) Tumor necrosis factor-α induces skeletal muscle insulin resistance in healthy human subjects via inhibition of Akt substrate 160 phosphorylation. Diabetes 54, 2939–2945 10.2337/diabetes.54.10.2939 16186396

[65] Cawthorn W.P. and Sethi J.K. (2008) TNF-alpha and adipocyte biology. FEBS Lett. 582, 117–131 10.1016/j.febslet.2007.11.051 18037376 PMC4304634

[66] Yang X., Zhang X., Heckmann B.L., Lu X. and Liu J. (2011) Relative contribution of adipose triglyceride lipase and hormone-sensitive lipase to tumor necrosis factor-α (TNF-α)-induced lipolysis in adipocytes. J. Biol. Chem. 286, 40477–40485 10.1074/jbc.M111.257923 21969372 PMC3220500

[67] Gerriets V., Goyal A. and Khaddour K. (2023) Tumor necrosis factor inhibitors. StatPearls, StatPearls Publishing 29494032

[68] Balsano R., Kruize Z., Lunardi M., Comandatore A., Barone M., Cavazzoni A. et al. (2022) Transforming growth factor-beta signaling in cancer-induced cachexia: from molecular pathways to the clinics. Cells 11, 2671 10.3390/cells11172671 36078078 PMC9454487

[69] Greco S.H., Tomkötter L., Vahle A.-K., Rokosh R., Avanzi A., Mahmood S.K. et al. (2015) TGF-β blockade reduces mortality and metabolic changes in a validated murine model of pancreatic cancer cachexia. PloS ONE 10, e0132786 10.1371/journal.pone.0132786 26172047 PMC4501823

[70] Liu D., Black B.L. and Derynck R. (2001) TGF-beta inhibits muscle differentiation through functional repression of myogenic transcription factors by Smad3. Genes Dev. 15, 2950–2966 10.1101/gad.925901 11711431 PMC312830

[71] Chen J.L., Walton K.L., Hagg A., Colgan T.D., Johnson K., Qian H. et al. (2017) Specific targeting of TGF-β family ligands demonstrates distinct roles in the regulation of muscle mass in health and disease. Proc. Natl. Acad. Sci. U.S.A. 114, E5266–E5275 10.1073/pnas.1620013114 28607086 PMC5495232

[72] Amirouche A., Durieux A.C., Banzet S., Koulmann N., Bonnefoy R., Mouret C. et al. (2009) Down-regulation of Akt/mammalian target of rapamycin signaling pathway in response to myostatin overexpression in skeletal muscle. Endocrinology 150, 286–294 10.1210/en.2008-0959 18801898

[73] Bahn Y.J., Wang Y., Dagur P., Scott N., Cero C., Long K.T. et al. (2024) TGF-β antagonism synergizes with PPARγ agonism to reduce fibrosis and enhance beige adipogenesis. Mol. Metab. 90, 102054 10.1016/j.molmet.2024.102054 39461664 PMC11570741

[74] Bou Matar D., Zhra M., Nassar W.K., Altemyatt H., Naureen A., Abotouk N. et al. (2025) Adipose tissue dysfunction disrupts metabolic homeostasis: mechanisms linking fat dysregulation to disease. Front Endocrinol (Lausanne) 16, 1592683 10.3389/fendo.2025.1592683 40630101 PMC12234313

[75] Alves M.J., Figuerêdo R.G., Azevedo F.F., Cavallaro D.A., Neto N.I., Lima J.D. et al. (2017) Adipose tissue fibrosis in human cancer cachexia: the role of TGFβ pathway. BMC Cancer 17, 190 10.1186/s12885-017-3178-8 28288584 PMC5348844

[76] Greco R., Qu H., Qu H., Theilhaber J., Shapiro G., Gregory R. et al. (2020) Pan-TGFβ inhibition by SAR439459 relieves immunosuppression and improves antitumor efficacy of PD-1 blockade. Oncoimmunology 9, 1811605 10.1080/2162402X.2020.1811605 33224628 PMC7657645

[77] Ling T., Zhang J., Ding F. and Ma L. (2023) Role of growth differentiation factor 15 in cancer cachexia (Review). Oncol. Lett. 26, 462 10.3892/ol.2023.14049 37780545 PMC10534279

[78] Li W., Moylan J.S., Chambers M.A., Smith J. and Reid M.B. (2009) Interleukin-1 stimulates catabolism in C2C12 myotubes. Am. J. Physiol. Cell Physiol. 297, C706–C714 10.1152/ajpcell.00626.2008 19625606 PMC2740393

[79] Herr S.M., Stalkopf D., Padaszus S., Herbst L.A., Dörrie A., Niedenthal R. et al. (2026) MK2/p38/p53 suppress basal IL-1β and non-canonical NF-κB signaling in macrophages. Int. J. Mol. Sci. 27, 3232 10.3390/ijms27073232 41977413 PMC13072840

[80] Rom O. and Reznick A.Z. (2016) The role of E3 ubiquitin-ligases MuRF-1 and MAFbx in loss of skeletal muscle mass. Free Radic. Biol. Med. 98, 218–230 10.1016/j.freeradbiomed.2015.12.031 26738803

[81] Laird B.J., McMillan D., Skipworth R.J.E., Fallon M.T., Paval D.R., McNeish I. et al. (2021) The emerging role of interleukin 1β (IL-1β) in cancer cachexia. Inflammation 44, 1223–1228 10.1007/s10753-021-01429-8 33907915 PMC8285330

[82] Hardee J.P., Montalvo R.N. and Carson J.A. (2017) Linking cancer cachexia-induced anabolic resistance to skeletal muscle oxidative metabolism. Oxid. Med. Cell Longev. 2017, 8018197 10.1155/2017/8018197 29375734 PMC5742498

[83] Bing C. (2015) Is interleukin-1β a culprit in macrophage-adipocyte crosstalk in obesity? Adipocyte 4, 149–152 10.4161/21623945.2014.979661 26167419 PMC4496963

[84] Agustsson T., Rydén M., Hoffstedt J., van Harmelen V., Dicker A., Laurencikiene J. et al. (2007) Mechanism of increased lipolysis in cancer cachexia. Cancer Res. 67, 5531–5537 10.1158/0008-5472.CAN-06-4585 17545636

[85] Xiong H., Ye J., Xie K., Hu W., Xu N. and Yang H. (2022) Exosomal IL-8 derived from lung cancer and colon cancer cells induced adipocyte atrophy via NF-κB signaling pathway. Lipids Health Dis. 21, 147 10.1186/s12944-022-01755-2 36581870 PMC9798689

[86] Frydelund-Larsen L., Penkowa M., Akerstrom T., Zankari A., Nielsen S. and Pedersen B.K. (2007) Exercise induces interleukin-8 receptor (CXCR2) expression in human skeletal muscle. Exp. Physiol. 92, 233–240 10.1113/expphysiol.2006.034769 17030560

[87] Callaway C.S., Delitto A.E., Patel R., Nosacka R.L., D'Lugos A.C., Delitto D. et al. (2019) IL-8 released from human pancreatic cancer and tumor-associated stromal cells signals through a CXCR2-ERK1/2 axis to induce muscle atrophy. Cancers (Basel) 11, 1863 10.3390/cancers11121863 31769424 PMC6966692

[88] VanderVeen B.N., Fix D.K. and Carson J.A. (2017) Disrupted skeletal muscle mitochondrial dynamics, mitophagy, and biogenesis during cancer cachexia: a role for inflammation. Oxid. Med. Cell Longev. 2017, 3292087 10.1155/2017/3292087 28785374 PMC5530417

[89] Bruun J.M., Lihn A.S., Madan A.K., Pedersen S.B., Schiøtt K.M., Fain J.N. et al. (2004) Higher production of IL-8 in visceral vs. subcutaneous adipose tissue. Implication of nonadipose cells in adipose tissue. Am. J. Physiol. Endocrinol. Metab. 286, E8–E13 10.1152/ajpendo.00269.2003 13129857

[90] Chait A. and den Hartigh L.J. (2020) Adipose tissue distribution, inflammation and its metabolic consequences, including diabetes and cardiovascular disease. Front Cardiovasc. Med. 7, 22 10.3389/fcvm.2020.00022 32158768 PMC7052117

[91] Kobashi C., Asamizu S., Ishiki M., Iwata M., Usui I., Yamazaki K. et al. (2009) Inhibitory effect of IL-8 on insulin action in human adipocytes via MAP kinase pathway. J. Inflamm. (Lond.) 6, 25 10.1186/1476-9255-6-25 19709445 PMC2746203

[92] Bilusic M., Heery C.R., Collins J.M., Donahue R.N., Palena C., Madan R.A. et al. (2019) Phase I trial of HuMax-IL8 (BMS-986253), an anti-IL-8 monoclonal antibody, in patients with metastatic or unresectable solid tumors. J. Immunother. Cancer 7, 240 10.1186/s40425-019-0706-x 31488216 PMC6729083

[93] López-Armada M.J., Riveiro-Naveira R.R., Vaamonde-García C. and Valcárcel-Ares M.N. (2013) Mitochondrial dysfunction and the inflammatory response. Mitochondrion 13, 106–118 10.1016/j.mito.2013.01.003 23333405

[94] Zhou W., Jiang Z.W., Tian J., Jiang J., Li N. and Li J.S. (2003) Role of NF-kappaB and cytokine in experimental cancer cachexia. World J. Gastroenterol. 9, 1567–1570 10.3748/wjg.v9.i7.1567 12854165 PMC4615506

[95] Singh S.K. and Singh R. (2022) Cytokines and chemokines in cancer cachexia and its long-term impact on COVID-19. Cells 11, 579 10.3390/cells11030579 35159388 PMC8834385

[96] Guo Q., Jin Y., Chen X., Ye X., Shen X., Lin M. et al. (2024) NF-κB in biology and targeted therapy: new insights and translational implications. Signal Transduct. Targeted Therapy 9, 53 10.1038/s41392-024-01757-9 PMC10910037 38433280

[97] Baker R.G., Hayden M.S. and Ghosh S. (2011) NF-κB, inflammation, and metabolic disease. Cell Metab. 13, 11–22 10.1016/j.cmet.2010.12.008 21195345 PMC3040418

[98] Mourkioti F. and Rosenthal N. (2008) NF-kappaB signaling in skeletal muscle: prospects for intervention in muscle diseases. J. Mol. Med. (Berl.) 86, 747–759 10.1007/s00109-008-0308-4 18246321 PMC2480606

[99] Zhang J., Zheng J., Chen H., Li X., Ye C., Zhang F. et al. (2022) Curcumin targeting NF-κB/ubiquitin-proteasome-system axis ameliorates muscle atrophy in triple-negative breast cancer cachexia mice. Mediators Inflamm. 2022, 2567150 10.1155/2022/2567150 35132306 PMC8817892

[100] Setiawan T., Sari I.N., Wijaya Y.T., Julianto N.M., Muhammad J.A., Lee H. et al. (2023) Cancer cachexia: molecular mechanisms and treatment strategies. J. Hematol. Oncol. 16, 54 10.1186/s13045-023-01454-0 37217930 PMC10204324

[101] Lv X. and Ding S. (2025) Unraveling the role of STAT3 in cancer cachexia: pathogenic mechanisms and therapeutic opportunities. Front. Endocrinol. 16, 1608612 10.3389/fendo.2025.1608612 PMC12283293 40704145

[102] Mengie Ayele T., Tilahun Muche Z., Behaile Teklemariam A., Bogale Kassie A. and Chekol Abebe E. (2022) Role of JAK2/STAT3 signaling pathway in the tumorigenesis, chemotherapy resistance, and treatment of solid tumors: a systemic review. J. Inflammation Res. 15, 1349–1364 10.2147/JIR.S353489 PMC8887966 35241923

[103] Wang B., Hsu S.H., Frankel W., Ghoshal K. and Jacob S.T. (2012) Stat3-mediated activation of microRNA-23a suppresses gluconeogenesis in hepatocellular carcinoma by down-regulating glucose-6-phosphatase and peroxisome proliferator-activated receptor gamma, coactivator 1 alpha. Hepatology 56, 186–197 10.1002/hep.25632 22318941 PMC3355233

[104] Gandhi A.Y., Yu J., Gupta A., Guo T., Iyengar P. and Infante R.E. (2022) Cytokine-mediated STAT3 transcription supports ATGL/CGI-58-dependent adipocyte lipolysis in cancer cachexia. Front Oncol. 12, 841758 10.3389/fonc.2022.841758 35785158 PMC9240385

[105] Attaix D., Ventadour S., Codran A., Béchet D., Taillandier D. and Combaret L. (2005) The ubiquitin-proteasome system and skeletal muscle wasting. Essays Biochem. 41, 173–186 10.1042/bse0410173 16250905

[106] Hughes D.C., Goodman C.A., Baehr L.M., Gregorevic P. and Bodine S.C. (2023) A critical discussion on the relationship between E3 ubiquitin ligases, protein degradation, and skeletal muscle wasting: it’s not that simple. Am. J. Physiol. Cell Physiol. 325, C1567–C1582 10.1152/ajpcell.00457.2023 37955121 PMC10861180

[107] Zhong G., Chang X., Xie W. and Zhou X. (2024) Targeted protein degradation: advances in drug discovery and clinical practice. Signal Transduct. Targeted Therapy 9, 308 10.1038/s41392-024-02004-x PMC11539257 39500878

[108] Choy E.H., De Benedetti F., Takeuchi T., Hashizume M., John M.R. and Kishimoto T. (2020) Translating IL-6 biology into effective treatments. Nat. Rev. Rheumatol. 16, 335–345 10.1038/s41584-020-0419-z 32327746 PMC7178926

[109] Wong M., Ziring D., Korin Y., Desai S., Kim S., Lin J. et al. (2008) TNFalpha blockade in human diseases: mechanisms and future directions. Clin. Immunol. 126, 121–136 10.1016/j.clim.2007.08.013 17916444 PMC2291518

[110] Gordon J.N., Trebble T.M., Ellis R.D., Duncan H.D., Johns T. and Goggin P.M. (2005) Thalidomide in the treatment of cancer cachexia: a randomised placebo controlled trial. Gut 54, 540–545 10.1136/gut.2004.047563 15753541 PMC1774430

[111] Flaherty S.E. 3rd, Song L., Albuquerque B., Rinaldi A., Piper M., Shanthappa D.H. et al. (2025) GDF15 neutralization ameliorates muscle atrophy and exercise intolerance in a mouse model of mitochondrial myopathy. J. Cachexia Sarcopenia Muscle 16, e13715 10.1002/jcsm.13715 39976232 PMC11840706

[112] Ruiz Garcia V., López-Briz E., Carbonell Sanchis R., Gonzalvez Perales J.L. and Bort-Marti S. (2013) Megestrol acetate for treatment of anorexia-cachexia syndrome. Cochrane Database Syst. Rev. 2013, Cd004310 10.1002/14651858.CD004310.pub3 23543530 PMC6418472

[113] Badowski M.E. and Yanful P.K. (2018) Dronabinol oral solution in the management of anorexia and weight loss in AIDS and cancer. Ther. Clin. Risk Manag. 14, 643–651 10.2147/TCRM.S126849 29670357 PMC5896684

[114] Schroeder E.T., Zheng L., Yarasheski K.E., Qian D., Stewart Y., Flores C. et al. (2004) Treatment with oxandrolone and the durability of effects in older men. J. Appl. Physiol. 96, 1055–1062 10.1152/japplphysiol.00808.2003 14578370

[115] Ji H., Ye T., Nie X., Chen Y., Li H., Chen S. et al. (2026) Circulating biomarkers: facilitating the diagnosis and treatment of cancer and other chronic diseases. In International Review of Cell and Molecular Biologyvol. 399, (Spada S. and Galluzzi L., eds), pp. 145–184, Academic Press, 50 Hampshire Street, 5th Floor, Cambridge, MA 02139, United States 10.1016/bs.ircmb.2025.05.001 41720563

[116] Bhol N.K., Bhanjadeo M.M., Singh A.K., Dash U.C., Ojha R.R., Majhi S. et al. (2024) The interplay between cytokines, inflammation, and antioxidants: mechanistic insights and therapeutic potentials of various antioxidants and anti-cytokine compounds. Biomed. Pharmacotherapy 178, 117177 10.1016/j.biopha.2024.117177 39053423

[117] Kadakia K.C., Hamilton-Reeves J.M. and Baracos V.E. (2023) Current therapeutic targets in cancer cachexia: a pathophysiologic approach. Am. Soc. Clin. Oncol. Educ. Book 43, e389942 10.1200/EDBK_389942 37290034 PMC11019847

[118] Al-Ibraheem A.M.T., Hameed A.A.Z., Marsool M.D.M., Jain H., Prajjwal P., Khazmi I. et al. (2024) Exercise-induced cytokines, diet, and inflammation and their role in adipose tissue metabolism. Health Sci. Rep. 7, e70034 10.1002/hsr2.70034 39221051 PMC11365580

[119] Griggs R.C., Kingston W., Jozefowicz R.F., Herr B.E., Forbes G. and Halliday D. (1989) Effect of testosterone on muscle mass and muscle protein synthesis. J. Appl. Physiol. (1985) 66, 498–503 10.1152/jappl.1989.66.1.498 2917954

[120] Tiidus P.M. (2011) Benefits of estrogen replacement for skeletal muscle mass and function in post-menopausal females: evidence from human and animal studies. Eurasian J. Med. 43, 109–114 10.5152/eajm.2011.24 25610174 PMC4261347

